# Efficient Noncollinear Second‐Harmonic Generation in Pb(In_1/2_Nb_1/2_)O_3_‐Pb(Mg_1/3_Nb_2/3_)O_3_‐PbTiO_3_ Crystals Through Domain‐Engineering

**DOI:** 10.1002/advs.202510153

**Published:** 2025-08-21

**Authors:** Xin Liu, Wenxu Huang, Kexin Song, Haisheng Guo, Weigang Zhao, Fusheng Qiu, Zhuo Xu, Fei Li, Xiaoyong Wei

**Affiliations:** ^1^ Electronic Materials Research Lab Key Lab of Education Ministry School of Electronic Science and Engineering Xi'an Jiaotong University Xi'an 710049 China; ^2^ National Key Laboratory of Science and Technology on Space Microwave China Academy of Space Technology Xi'an 710100 China

**Keywords:** domain engineering, ferroelectrics, nonlinear optics, phase matching

## Abstract

Achieving broadband and effective frequency conversion through non‐collinear phase matching (PM) is of significant interest due to its potential applications in all‐optical signal processing. To overcome the stringent requirements of the quasi‐phase‐matching condition, such as the period width, polarization, and incident direction, perovskite‐type ferroelectric materials with natural ferroelectric domains offer advantages for achieving broadband second‐harmonic generation (SHG). However, the random distribution of lattice vectors and scattering at domain walls not only causes substantial scattering losses, limiting conversion efficiency, but also complicates detection and application. In this study, we present a new type of nonlinear photonic crystal by constructing a domain structure with a regular distribution of polarization vectors in a Pb(In_1/2_Nb_1/2_)O_3_‐Pb(Mg_1/3_Nb_2/3_)O_3_‐PbTiO_3_ (PIN‐PMN‐PT) crystal. This structure enables SHG with increased efficiency (6 × 10^−5^) at fundamental wavelengths ranging from 1064 to 1340 nm without the need for angle or temperature tuning. Additionally, this unique domain structure facilitates the spontaneous deviation of the SHG emission direction from the propagation direction of fundamental‐frequency light (FL), thus eliminating detection and application challenges. This work presents a new method of achieving broadband and efficient noncollinear SHG using domain engineering, and also pushes the boundaries of frequency conversion techniques in ferroelectrics.

## Graphical abstract

This study introduces a domain‐engineered PIN‐PMN‐PT ferroelectric crystal that enables efficient broadband second harmonic generation (SHG) without angle or temperature tuning. By engineering a “2T” domain structure, high SHG efficiency is achieved by noncollinear phase matching. This work presents a new method of achieving broadband and efficient noncollinear SHG using domain engineering, pushing the boundaries of frequency conversion techniques in ferroelectrics.

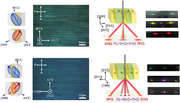

## 1 Introduction

Nonlinear optical crystals play a critical role in nonlinear optical frequency conversion applications in information processing and telecommunication networks.^[^ ^1^, ^2^ ^]^ Among the key parameters influencing the effectiveness of nonlinear optical devices, the most important is the nonlinear conversion efficiency, which is significantly affected by factors such as pump energy, inherent nonlinear‐optical properties, and phase matching (PM) conditions. To achieve efficient frequency conversion, a large effective second‐order nonlinearity coefficient, along with optimal PM conditions, is typically required.

For crystals with low birefringence, where PM cannot be achieved through angle selection, quasi‐phase matching (QPM) has been proposed as an alternative approach.^[^ ^3^, ^4^ ^]^ By applying an electric field to a sample with appropriately patterned electrodes, the polarization vector of the ferroelectric domains, and thus the sign of the nonlinear coefficient, can be selectively inverted, effectively providing additional reciprocal vectors (G) to compensate for phase mismatches.^[^ ^5^, ^6^, ^7^, ^8^ ^]^ In addition to external electric field poling, other methods, such as chemical indiffusion,^[^ ^9^ ^]^ scanning force microscopic poling, electron‐beam poling and optical poling^[^ ^10^, ^11^, ^12^ ^]^ have been developed for fabricating periodically poled single crystals and modifying surface domain structures in ferroelectric materials.

QPM has expanded the range of materials suitable for nonlinear optical devices, however, strict control over the period width and duty cycle of the ferroelectric domains is essential to satisfy the requirements of the fundamental light (FL) wavelength and various nonlinear optical processes. For example, achieving efficient frequency doubling for FL at 1064 nm in MgO‐doped periodic poled lithium niobate (PPLN) requires a period width of 6.9 µm, whereas 12 µm is needed for effective second harmonic generation (SHG) at 1320 nm. Thus, to realize effective broadband frequency conversion, periodic domain structures with varying periods or temperature tuning is needed.^[^ ^13^ ^]^ However, this approach poses challenges in fabrication, and may lead to larger device sizes. Additionally, efficient nonlinear conversion imposes strict directional constraints on the cutting orientation and incident light polarization of the crystal. To overcome such limitations, materials with random domains have been selected to achieve broadband PM. This approach involves nonlinear PM mechanisms that enable the conversion of FL into higher‐order harmonics over a broad spectrum. In 2008, Molina et al.^[^ ^14^ ^]^ demonstrated that different planar and conical nonlinear processes across a wide spectral range can be achieved in samarium barium niobate (SBN) crystals, which can function as multiple‐wavelength converters. In 2014, Sheng et al.^[^ ^15^ ^]^ achieved broadband SHG in as‐grown Ca_0.28_Ba_0.72_Nb_2_O_6_ (CBN) crystals with randomly sized ferroelectric domains and demonstrated the ability of CBN crystals to achieve high nonlinear optical conversion in the fundamental wavelength range of 1.35–1.46 µm. In 2016, broadband petal‐like SH patterns were observed in the naturally grown Ba_0.77_Ca_0.23_TiO_3_ (BCT) crystals.^[^ ^16^, ^17^ ^]^ Later, Chang Li et al. demonstrated the rich reciprocal vectors provided by 3D naturally grown KTa_1‐x_Nb_x_O_3_ perovskite, in which broadband SHG was also observed.^[^ ^18^ ^]^

Preliminary work has mainly focused on the nonlinear PM mechanism and the application of naturally grown ferroelectric crystals with randomly distributed domain structures. Although the rich domain structures of these materials provide abundant PM vectors, certain limitations have been identified. First, the random distribution of inverted lattice vectors and nonlinear scattering at domain boundaries (domain walls) results in substantial scattering losses, hindering efforts to improve nonlinear optical conversion efficiency. Additionally, the SHG patterns observed in naturally grown multidomain samples vary in shape depending on the lattice type, often appearing as circular, linear, or dot matrix configurations. As a result, it becomes crucial to develop effective methods for filtering high‐energy FL in practical applications, further complicating implementation.

Domain engineering has proven to be an effective method for creating stable, tailored domain patterns to modulate electro‐optical and nonlinear optical properties.^[^ ^19^, ^20^, ^21^, ^22^ ^]^ In this study, we selected 0.21PIN‐(0.79−*x*) PMN‐*x*PT (*x* = 0.38) crystals due to their promising nonlinear optical properties^[^ ^20^ ^]^ and high stability under temperature and electric field conditions. Two types of PM were achieved in this domain‐engineered tetragonal PIN‐PMN‐PT single crystal. The light‐field distribution of the SH light was concentrated and spontaneously deviated from the FL propagation direction. Furthermore, the conversion efficiency reached 6 × 10^−5^ at fundamental wavelengths ranging from 1064 to 1340 nm, surpassing the conversion efficiency achieved in naturally grown perovskite crystals within this range. This study introduces a new type of photonic crystal and advances the QPM technique, offers a new platform for nonlinear optical applications.

## 2 Results and Discussion

### 2.1 Characterization of the Domain Structure of PIN‐PMN‐PT

Recently, a layered domain structure was formed in a rhombohedral PIN‐PMN‐PT single crystal by poling along the [011] direction, which enabled high transparency and a large electro‐optic coefficient.^[^ ^23^, ^24^, ^25^, ^26^ ^]^

**Figure 1:**
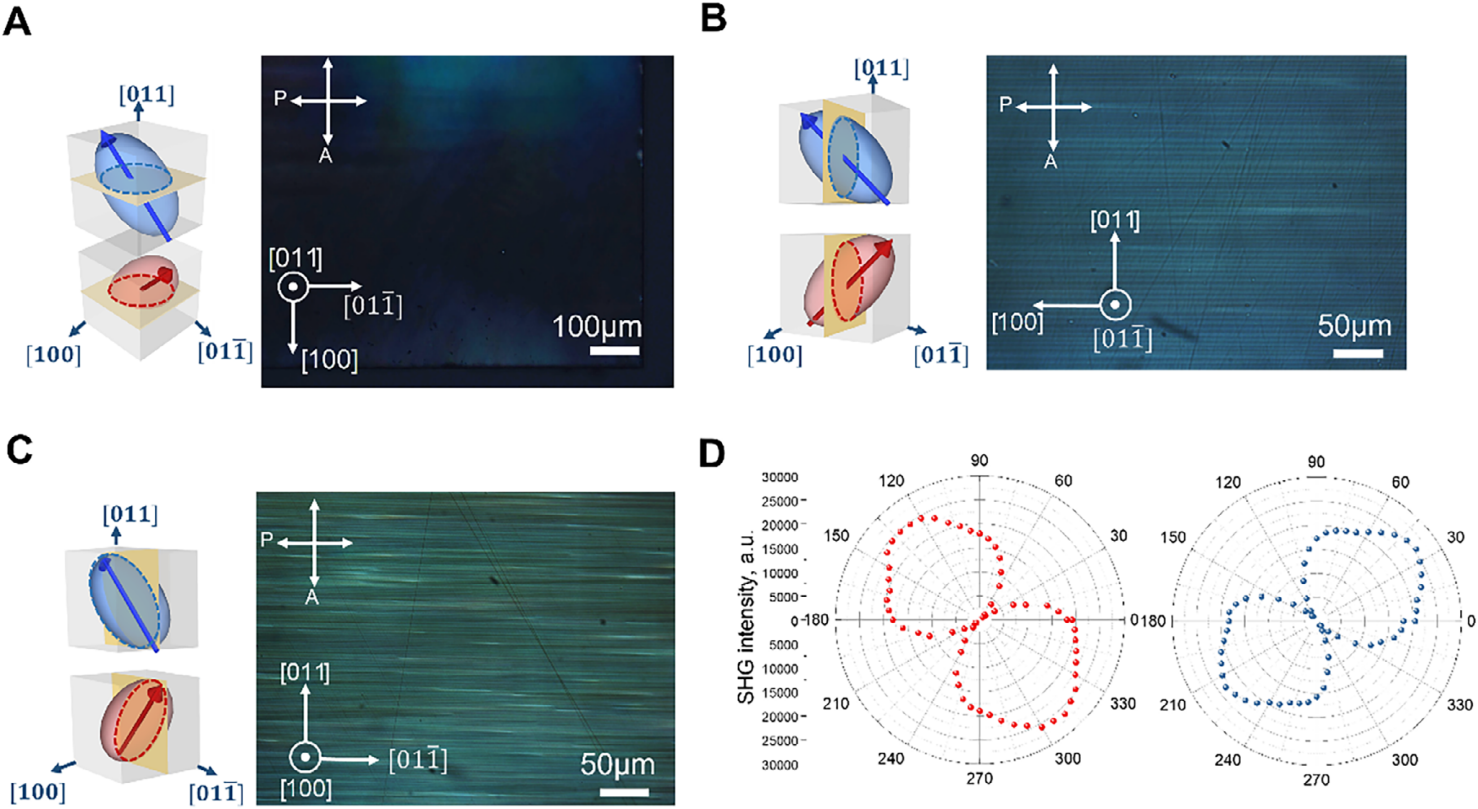
Domain patterns and polarization orientation in a [011]‐poled tetragonal PIN‐PMN‐PT crystal. A) B,C) show polarized light microscope (PLM) images and schematic diagrams of the optical indicatrix for both domains projected onto the (011), $\left(01\bar{1}\right)$, and (100) planes. As illustrated, the principal axes of the refractive indices in both the [001] and $\left[01\bar{1}\right]$ domains projected onto the (011) or (100) plane are identical. D) shows the angular dependence of the non‐analyzed SHG intensity on the incident light polarization of adjacent single domain region.

In this study, the tetragonal PIN‐PMN‐PT single crystals were first poled along the [011] direction to form a regular domain structure for subsequent analysis. In naturally grown tetragonal PIN‐PMN‐PT, the spontaneous polarization vectors aligned with one of the six equivalent <001> directions. After poling along the [011] direction, only two domain variants with polarization vectors along [010] and [001] exist, forming a relatively simple “2T” engineered domain structure (Figure S1, Supporting Information).^[^ ^27^ ^]^ As shown in **Figure** **1**A, the projections of the principal axes of the optical indicatrix for both the [010] and [001] domains on the (011) plane are identical, indicating that the refractive indices along the [011] direction remain unchanged. Additionally, because the domain walls of the “2T” structure are parallel to the (011) plane, the polarized light microscopy images of the (011) face are uniformly dark. The projections of the optical indicatrix principal axes differed on the (100) plane and were identical on the $\left(01\bar{1}\right)$ plane (Figure 1B,C). Moreover, lamellar domain configurations were observed on both the (100) and $\left(01\bar{1}\right)$ faces. This observation contrasts with that of the [011]‐poled rhombohedral PIN‐PMN‐PT crystals, where lamellar domain configurations with smaller domain sizes are typically observed only on (01) face.

We measured the intensity of SH light containing all polarization states with the incident light polarization angle θ_ω_ set parallel to the [011] direction of the sample (Figure 1D). Due to the different orientations of the polarization vectors in adjacent domains, the SH light exhibits significant anisotropy because of the anisotropy of nonlinear optical coefficient. Additional detailed analyses are provided in the Supporting Information. The minimal SH signal was observed at θ_ω_ = 94° and 274° in one single domain region, while the minimal SH signal was observed at θ_ω_ = 0° and 180° in the adjacent domain. The results show a 90° phase shift in the SHG intensity between the two domain areas, confirming the stable existence of the “2T” domain structure.

### 2.2 Linear Optical Performance

**Figure 2:**
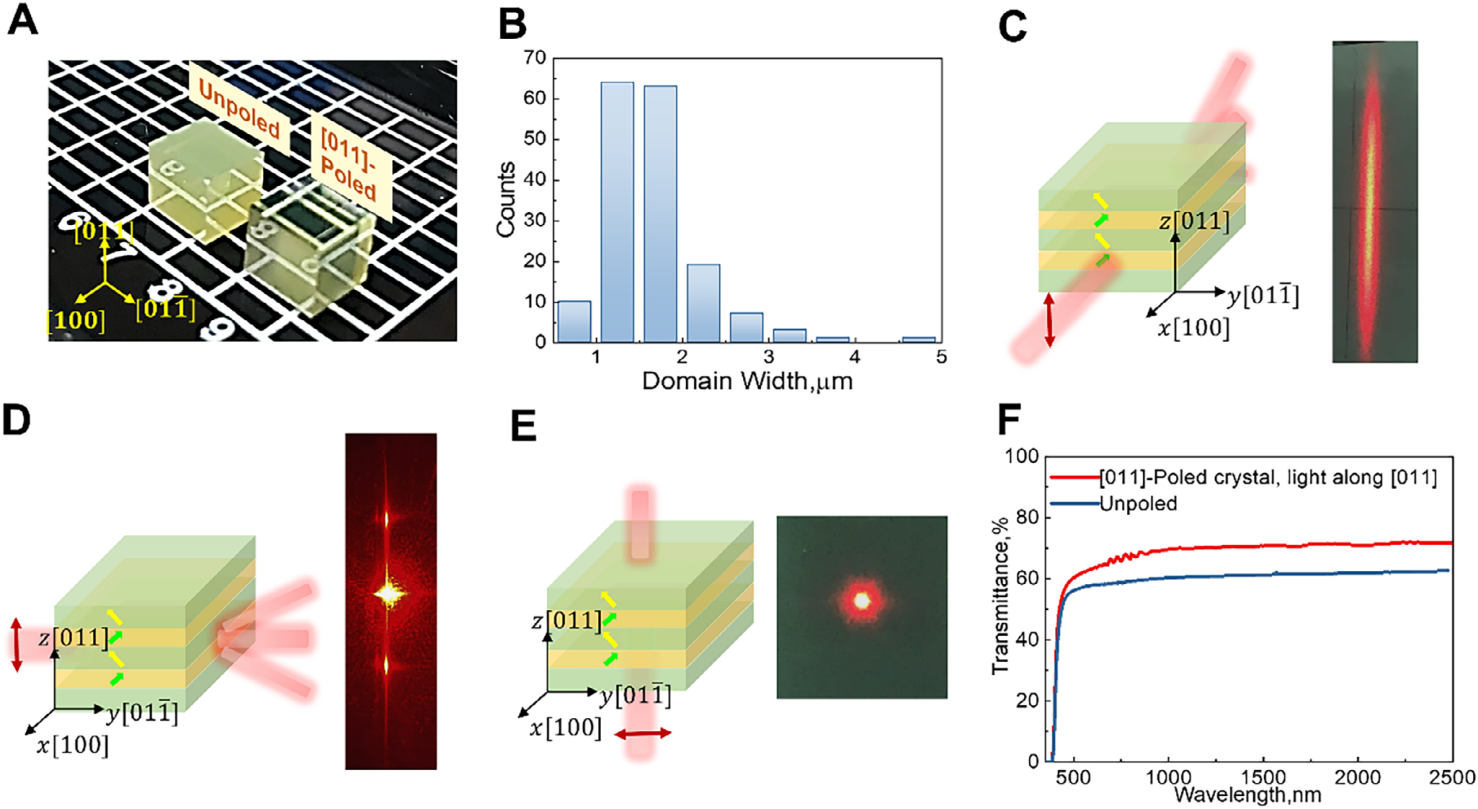
Linear optical properties of domain‐engineered PIN‐PMN‐PT crystals poled along the [011] direction. A) Image of poled PIN‐PMN‐PT single crystals displaying the major faces: (100), ($01\bar{1}$), and (011). An unpoled crystal is shown for comparison. B) Distribution of the domain width in a 0.5 mm sample slice from Figure 1. C–E) Output spot of a Gaussian beam propagating along the [100], [$01\bar{1}$] and [011] directions of the crystal, respectively. F) Optical transmission spectra of the [011]‐poled sample over the wavelength range of 400–2500 nm.

**Figure** **2**A shows the image of a poled tetragonal PIN‐PMN‐PT single‐crystal. For clarity, we denote the *x*, *y*, and *z*‐axes as [100], [$01\bar{1}$], and [011], respectively. In rhombohedral samples, high transparency can be achieved on both (100) and (011) faces, where the optical indicatrix on both sides of the domain walls exhibit identical projections.^[^ ^28^ ^]^ However, in tetragonal samples, the average domain size is significantly larger than that in rhombohedral samples, with an average domain size of ≈1–2 µm (Figure 2B). Transparency depends not only on the alignment of the optical indicatrix but also on factors such as the domain wall width and domain size, which result in light deflection. Despite having similar projections of the optimal indices, the ($01\bar{1}$) plane appeared opaque due to its larger domain size and relatively thick domain walls. Consequently, only high transmittance can be expected from the (011) plane.

Figure 2C–E show the schematic diagrams and real footprints of a Gaussian beam passing through the crystal when propagating along the *x*, *y*, and *z*‐axes, respectively. The incident light was polarized along the *z* direction. In Figure 2E, the Gaussian beam retains its shape when propagating along the *z*‐axis, however, severe deflection occurs when the light travels along the *x* or *y*‐axes (Figure 2C,D).

As shown in Figure S2 (Supporting Information), the morphologies of the outgoing light spots and their polarizations changed when the polarization direction of the incident light was altered, which was affected by the relationship between the refractive indices and domain wall structures. Optical transmittance along the [011] direction (Figure 2F) was measured before and after poling. Over the wavelength range of 500–2500 nm, a 20% increase in transmissivity was achieved through domain‐engineering optimization.

**Figure 3:**
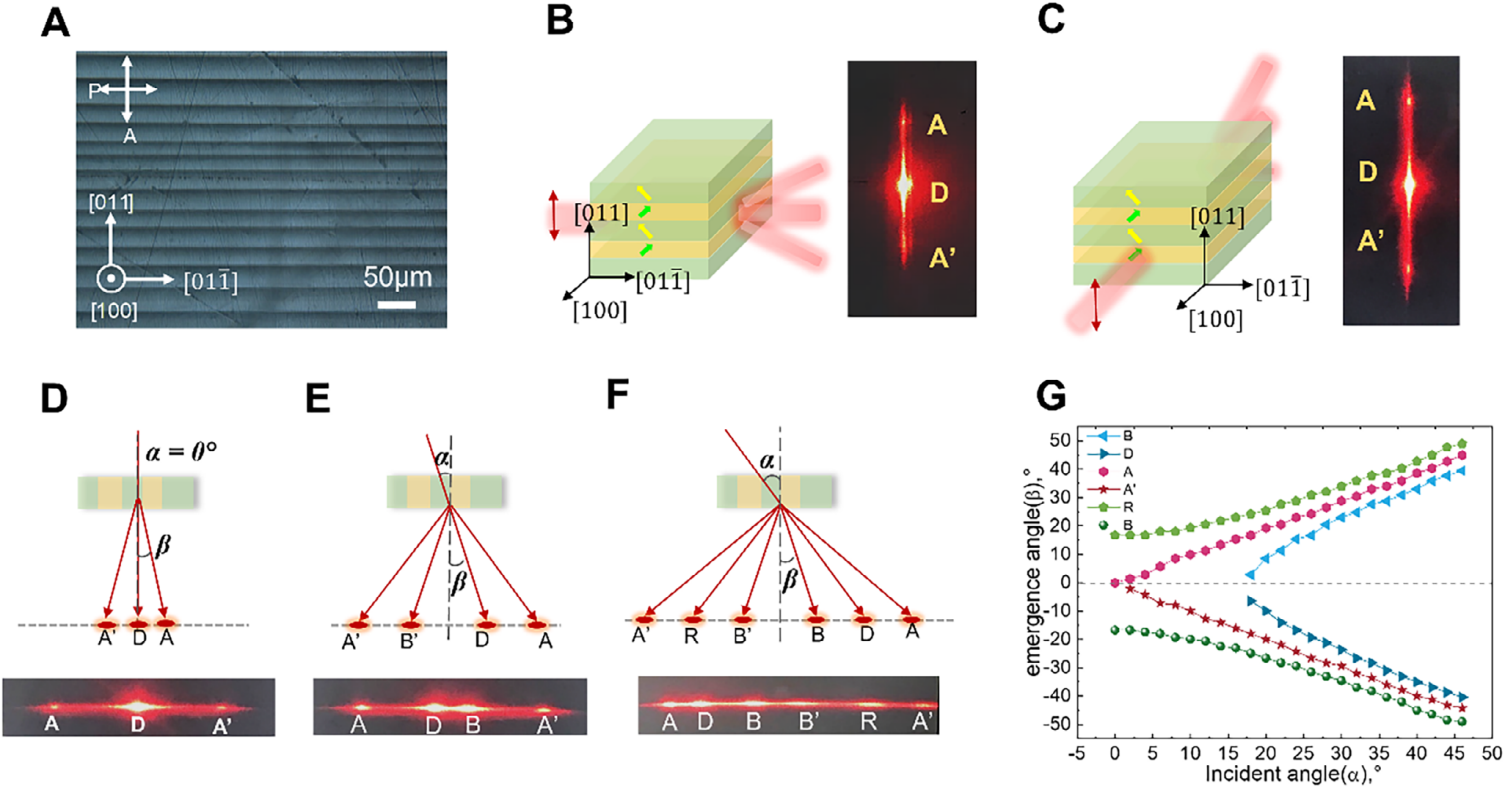
Different deflection phenomena in [011]‐poled PIN‐PMN‐PT crystals with a domain size of 15 µm for beam propagating along [100]. A) PLM image of the [011]‐poled tetragonal PIN‐PMN‐PT crystal with a domain size of 15 µm. B,C), Output spot of a Gaussian beam propagating along the [100] and [$01\bar{1}$] directions of the crystal, respectively. D–F), Schematic diagram and output spot of a Gaussian beam propagating along [100] as the incident angle increases, respectively. G) Variations in the angles of the deflected beams with respect to the incident angle.

In order to analyze the origin of light deflection in the sample with layered domain structure, we prepared samples with a domain size of 15 µm by orthogonal poling method.^[^ ^19^ ^]^ We found that the deflection behavior of the domain‐engineered PIN‐PMN‐PT single crystal was influenced not only by the polarization direction of the incident beam, but also by the domain size. As shown in **Figure** **3**A–C, a sample with a domain size of 15 µm exhibits distinct deflection characteristics to a smaller domain sample (e.g., 1–2 µm, Figure 2) when light propagates along the [$01\bar{1}$] and [100] directions.

The deflection behavior also depends on the incident angle of the laser beam. When the laser beam propagates in the [100] direction, as the incident angle α gradually increases, the number of scattered spots increases from three to six. Figure 3D–F shows the scattered beam patterns obtained at different incident angles (α = 0°, α = 5°, and α = 25°, respectively). Three types of deflected beams‐referred to as AA’, DR, and BB’ can be observed. Figure 3G shows the variation of the deflected angles of AA’, DR, and BB’ with respect to the incident angle (*α*) for the domain walls. In particular, when the incident angle α<α*_cri1_* (α*_cri1_* = 18°), only three spots (D, A, and A’) are observed. In the intermediate range α*_cri1_* ≤ α ≤ α*_cri2_*, where α*_cri2_* = 20°, four distinct spots (D, R, A, and A’) emerge. Beyond the second critical angle (α>α*_cri2_*), six resolvable spots are detected due to the refractions on the incident sample face, reflections and refractions on the domain walls, and refractions on the exit sample face.^[^ ^29^, ^30^, ^31^ ^]^

### 2.3 Nonlinear Optical Performance

**Figure 4:**
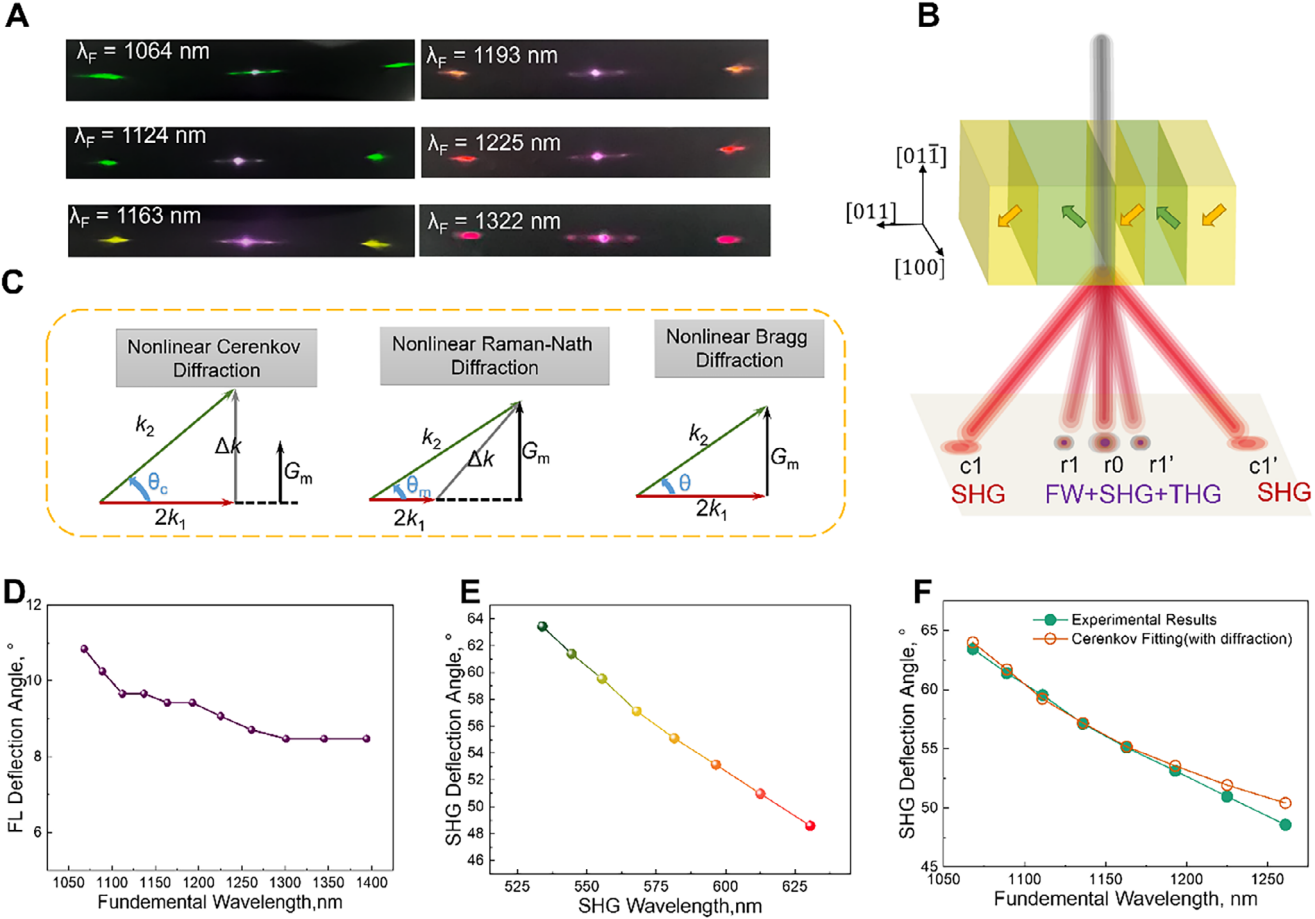
SHG and PM in the [$01\bar{1}$]‐cut PIN‐PMN‐PT sample with a “2T” domain structure. A) Output spot while FL propagates along the [$01\bar{1}$] direction. B) Schematic of the SHG and THG processes originating from the “2T” domain structure. C) Three types of nonlinear PM processes. D,E) Deflection angles of the FL (r_1_) and the SHG spot (c_1_) on the side, respectively. F, Fitting by the Čerenkov nonlinear PM equation.

First, the fundamental light is incident into the *y*‐plane $\left(01\bar{1}\right)$ with the polarization parallel to *z*‐axis ([011]), and the thickness of the sample size is 1.2 × 1.2 × 5 mm ([100] ×[$01\bar{1}$] × [011]). Far‐field patterns of the SH light were projected onto a screen and recorded using a camera.The SHG patterns shown in **Figure** **4**A were collected while tunning the fundamental wavelength (λ_ω_) from 1064 to 1322 nm. When the wavelength of the fundamental wave varied between 1124 and 1225 nm, the three spots in the middle appeared purple (Figure 4B), indicating that higher‐order noncollinear processes occurred inside the crystal within this wavelength range.

To achieve efficient non‒collinear SH generation, the following QPM condition must be satisfied

(1) $$k_{2}=2k_{1}+G_{m}$$

The PM relationships of nonlinear diffraction can be decomposed into longitudinal and transverse components, *k*_2_ *cos*θ_*c*_ = 2*k*_1_ and *k*_2_ *sin*θ_*m*_ = *G_m_* + Δ*k_x_*, respectively. In the case of the periodic modulation, $G_{m}=\frac{2\pi m}{\Lambda }$, where *m* is an integer representing the so called Raman–Nath nonlinear diffraction order and Λ is the periodicity of the domains.

Three distinct noncolinear PM types may be found in crystals (Figure 4C). If the frequency conversion process satisfies only the longitudinal PM condition, the generated SH beam is termed Čerenkov SH. Čerenkov SH light propagates at an angle defined by

(2) $$\theta_{c}=\arccos\left(\frac{2k_{1}}{k_{2}}\right)$$

If the nonlinear optical process satisfies only the transverse PM condition, the generated SH beam is termed nonlinear Raman–Nath SH and is analogous to the standard Raman–Nath diffraction in linear optics. Nonlinear Bragg diffraction is referred to simultaneously both transverse and longitudinal PM conditions.

To differentiate between the various nonlinear PM processes, the deflection angles of the three central spots and the sideband SHG spots were measured (Figure 4D,E). When the FL wavelength spans from 1050 to 1400 nm, the deflection angle of the central SH spots (r_1_ and r_1_’) gradually decreases from 11° to 9°. This behavior mirrors that observed in standard Raman–Nath diffraction in linear optics (Figure 3G). Therefore, these SH beams are referred to as nonlinear Raman–Nath SH beams.

The deflection angle of the SHG light with a larger deflection angle (c_1_ and c_1_’) decreased from 64° to 48° within the FL wavelength range of 1064–1322 nm. The fitting clearly revealed that these angles were consistent with the Čerenkov PM conditions, indicating that this SHG light was generated under the Čerenkov PM conditions (Figure 4F). We can see that the intensity of the Raman–Nath SH signal from the random domains is much weaker than that generated through nonlinear interactions under the Čerenkov PM condition.

**Figure 5:**
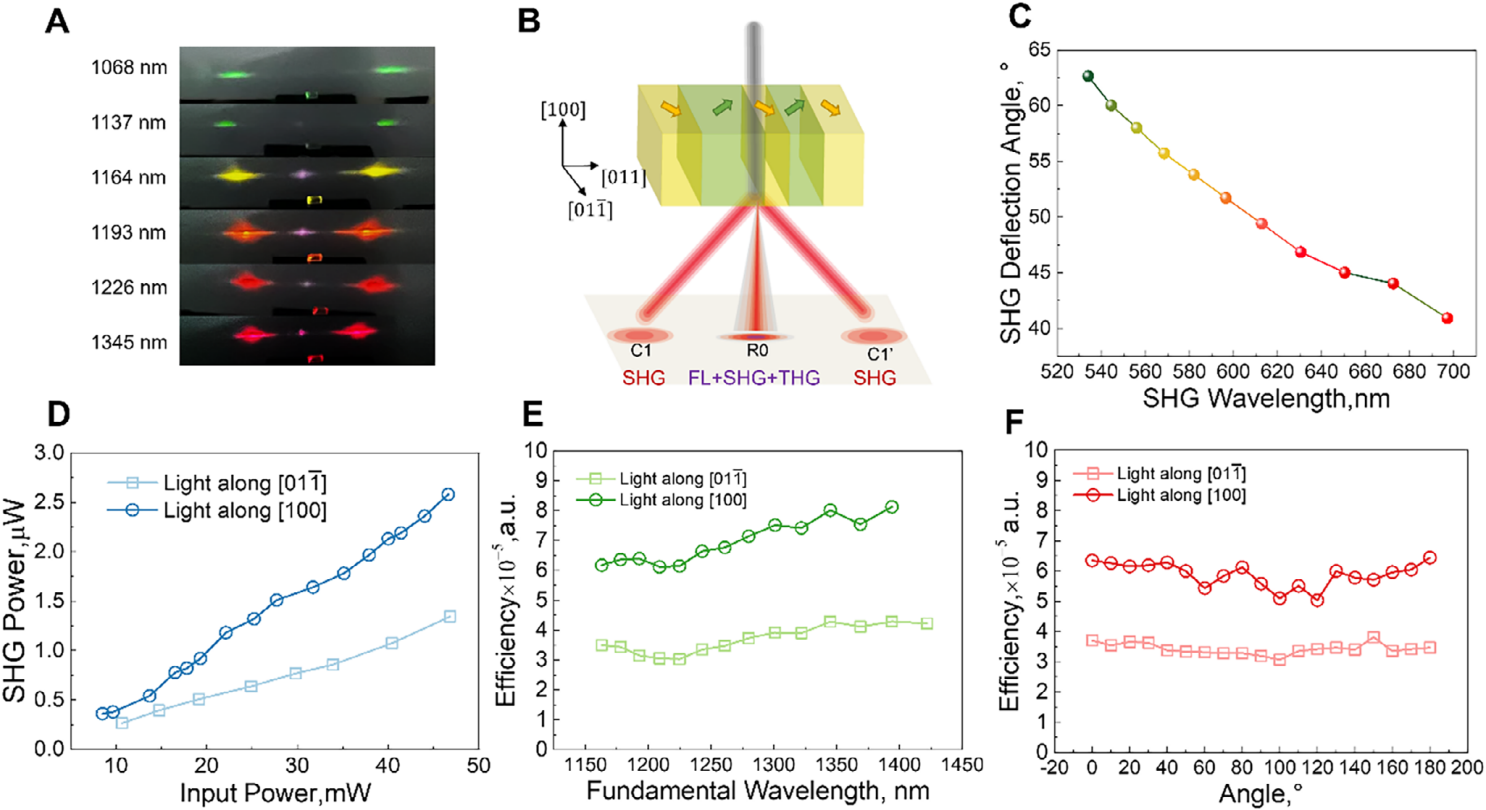
SHG processes in the [100]‐cut sample and comparison of the SH performance in the $\left[01\bar{1}\right]$ and [100]‒cut samples with respect to the input power, fundamental wavelength, and polarization direction. A) Output spot while FL propagates along the [$\bar{1}00$] direction. B) Schematic of the SHG process originating from the “2T” domain structure. C) Deflection angle of the Čerenkov spot on the side (C_1_). D) Dependence of the output power of spot C_1_ on the pump power. E) Dependence of the conversion efficiency of spot C_1_ on the fundamental wavelength. F) Dependence of the conversion efficiency of SH light (C_1_) on polarization angle at the fundamental wavelength of 1300 nm and a pump power of 30 mW.

When the laser beam was incident along the *x*‐axis ([100]), the SHG processes differed significantly from those observed in the *y*‐cut ($\left[01\bar{1}\right]$) sample. As shown in **Figure** **5**A, when the FL wavelength varied from 1068 to 1345 nm, a weak SH signal was observed in the [00$\bar{1}$] direction, and two SH spots with a broad angular intensity distribution were symmetrically distributed. The optical field distribution of the infrared FL is similar to that of visible light, as shown in Figure 2D. Figure 5B shows the schematic of the SHG or THG process originating from the “2T” domain structure.

The deflection angle of the sideband SH light (C_1_ and C_1_’) decreased from 63° to 47° when the fundamental wavelength was varied from 1064 to 1320 nm, aligned with the Čerenkov PM condition (Figure 5C). The relationship between the incident fundamental power at 1226 nm and the SHG power of Čerenkov light (c_1_ and C_1_ spots) for both cut samples was measured (Figure 5D). The SHG power increases quadratically with the incident fundamental power when it exceeds the threshold, which is consistent with the QPM theory. At an input power of 46.6 mW, the SHG power reached 25.8 µW for light incident along the [100] direction resulting in a conversion efficiency of 5.5 × 10^−5^, which was two times greater than that of the previous nonlinear beam‐shaping experiments. For incident light along the $\left[01\bar{1}\right]$ direction, the conversion efficiency was 2.8 × 10^−5^ at an input power of 46.8 mW.

The conversion efficiency of SH light as a function of FL wavelength was also investigated (Figure 5E). Unlike conventional photonic crystals with fixed periodicity, no significant peak was observed in the SH signal, indicating that effective PM over a wide frequency range can be achieved in PIN‐PMN‐PT by domain engineering. When the polarization direction of FL was altered, the emission characteristics of the SH spots did not undergo significant changes, in contrast to the behavior observed in the linear optical experiments (Figure S3, Supporting Information). The polarization angle of the FL relative to the optical axis was carefully adjusted, with the input power maintained at ≈30 mW. Notably, there was no substantial variation in the frequency conversion efficiency when the polarization direction of the FL was varied (Figure 5F). Thus, efficient broadband SHG was achieved in domain‐engineered PIN‐PMN‐PT crystals. Furthermore, we measured the conversion efficiency when the laser beam was incident along the *z*‐axis ([011]), the SHG beam can remain as a circular spot, indicating good alignment of the domain wall, however, the conversion efficiency is much lower (≈1.5 × 10^−5^) than that in the other two directions (Figure S4, Supporting Information).

## 3 Conclusion

After poling along the [011] direction, a tetragonal PIN‐PMN‐PT crystal can form a distinct domain structure with alternating orientations of adjacent domains, but variable domain sizes. This crystal exhibits distinct linear and nonlinear optical behaviors owing to its unique ferroelectric domain structure. Different beam‐deflection phenomena correspond to varying domain sizes and incident angles can be found, which arise from birefringence and domain‐wall diffraction. Additionally, the engineered domain structure in the PIN‐PMN‐PT enables the simultaneous occurrence of the two types of PM over a wide wavelength range. The key factor in broadband SHG is that the engineered domain structure satisfies noncollinear phase matching (Raman–Nath and Čerenkov) schemes. To be more specific, the peripheral spots, situated relatively far from the fundamental beam at both sides of the diffraction pattern is Čerenkov radiation originating from the steep change of the second‐order (χ^2^) nonlinearity across the domain wall. Since the longitudinal PM condition is not related to the size of the domains, broadband SHG can be expected. As to the central diffraction spots, which is called nonlinear Raman–Nath diffraction, the randomness of the domain pattern can provide various extra polarization vectors and meet the vertical PM condition over a wide wavelength range. As a result, efficient simultaneous generation of SH and TH was achieved. Compared to that of previous photonic crystals,^[^ ^17^, ^18^, ^22^ ^]^ the normalized internal conversion efficiency was higher, and the far‐field distribution of the SH was more concentrated in the PIN‐PMN‐PT photonic crystals. This noncollinear SHG phenomenon in the PIN‐PMN‐PT crystal with a layered domain structure differs from the results reported for crystals with similar domain structures,^[^ ^14^, ^16^ ^]^ indicating that the SHG performance originates not from the domain walls but from the additional vector provided by the engineered domain structure.

In conclusion, this study introduces a domain‐engineered PIN‐PMN‐PT ferroelectric crystal that enables efficient broadband SHG without angle or temperature tuning. By engineering a regular “2T” domain structure through [011] poling, the study achieves high SHG efficiency (up to 6 × 10^−5^) in tetragonal PIN‐PMN‐PT crystals across a wide range of fundamental wavelengths (1064–1340 nm). Moreover, the spontaneous deflection of SH light away from the pump beam path, eliminating challenges in SHG detection and application. We believe that our study makes a significant contribution to the literature and fabrication constraints typically associated with quasi‐phase matching. This work presents a new method of achieving broadband and efficient noncollinear SHG using domain engineering, pushing the boundaries of frequency conversion techniques in ferroelectrics.

## 4 Experimental Section

### Sample preparation

Pb(In_1/2_Nb_1/2_)O_3_‐Pb(Mg_1/3_Nb_2/3_)O_3_‐PbTiO_3_ crystals were grown using the modified Bridgman technique at Xi'an Jiaotong University. The crystal was cut along the desired orientation using X‐ray diffraction (XRD); the [100], [$01\bar{1}$], and [011] directions were assigned as *x*, *y*, and *z* axes, respectively.

The crystal was poled using a homemade setup consisting of a sample holder immersed in a silicone oil bath and a high‐voltage source (XTECH HV‐20 KV). Before poling, the samples were cut to the desired size and annealed at 700 °C for 5 h under short‐circuit conditions. The samples were then slowly cooled to room‐temperature to reduce internal stress. PIN‐PMN‐PT single crystals with different domain sizes were obtained by using orthogonal poling method. The orthogonal poling method mainly contains two steps. First, poling the tetragonal PIN‐PMN‐PT sample with an electric field (*E_1_*) of double coercive field along [011] direction at room‐temperature, the dwelling time is 5 min. Second, applying an electric field (*E_2_*) on the “2T” sample along the [$0\bar{1}1$] direction. In the experiment, the intensity of *E_2_* is 1/5 of coercive field. The longer the electric field was applied, the larger the size of the domains. The [011]‐poled PIN‐PMN‐38PT with domain size of 1–2 µm was used for SHG measurements and the sample size was 1.2 mm × 1.2 mm × 5mm ([100] ×[$01\bar{1}$] × [011]).

### Domain Characterizations

Optical observations of the domain structure were performed using a polarized light microscope (PLM) (OLYMPUS BX51, Japan) with a 0/90° crossed polarizer/analyzer pair. Second‐harmonic generation (SHG) microscopy and polarimetry were conducted using a modified Witec Alpha 300S confocal Raman microscope equipped with a 10 nm resolution XYZ piezo‐translation stage.

### Characterization of Nonlinear Optical Performance

An optical parametric oscillator (OPO, Litron) was used as the pump source. The maximum pulse energy of the incident laser was ≈63 mJ with a repetition rate of 10 Hz. The incident laser was focused by a convex lens with a focal length of 25 mm, and the radius of the focused spot was ≈1 mm, which was smaller than that of the sample but larger than that of the domains, where the diffraction effect cannot be neglected. Although the light generated by SHG was spontaneously separated from the FL, we still used a low‐pass filter in front of the detector to prevent interference from the fundamental beam.

## Conflict of Interest

The authors declare no conflict of interest.

## Supporting information



Supporting Information

## Data Availability

The data that support this study are available from the first author and corresponding authors upon reasonable request.
