# Supplementary material for: Efficient Noncollinear Second‐Harmonic Generation in Pb(In1/2Nb1/2)O3‐Pb(Mg1/3Nb2/3)O3‐PbTiO3 Crystals Through Domain‐Engineering
Source: Adv Sci (Weinh). 2025 Aug 21;12(41):e10153. doi: 10.1002/advs.202510153 (PMC12591212; doi:10.1002/advs.202510153)
Supplement: Supplementary file 1 — Supporting Information [file ADVS-12-e10153-s001.docx]

**Supporting Information for**

Efficient Noncollinear Second-Harmonic Generation in Pb(In_1/2_Nb_1/2_)O_3_-Pb(Mg_1/3_Nb_2/3_)O_3_-PbTiO_3_ Crystals Through Domain-Engineering

Xin Liu^1*^, Wenxu Huang^1^, Kexin Song^1^, Haisheng Guo^1^, Weigang Zhao^2^, Fusheng Qiu^1^, Zhuo Xu^1^, Fei Li^1^*, Xiaoyong Wei^1^*

^1^Electronic Materials Research Lab, Key Lab of Education Ministry, School of Electronic Science and Engineering, Xi’an Jiaotong University, Xi’an, 710049, China

^2^National Key Laboratory of Science and Technology on Space Microwave, China Academy of Space Technology (Xi'an), 710100, China

*Corresponding authors. E-mail: Xin Liu ([eudoraliu@xjtu.edu.cn](mailto:eudoraliu@xjtu.edu.cn)), Fei Li (ful5@xjtu.edu.cn), Xiaoyong Wei (wdy@mail.xjtu.edu.cn)

Keywords: Ferroelectrics; Domain Engineering; Phase Matching; Nonlinear optics;

1. **Experimental section**
   1. **Sample preparation**

The Pb(In_1/2_Nb_1/2_)O_3_-Pb(Mg_1/3_Nb_2/3_)O_3_-PbTiO_3_ crystal was grown using a modified Bridgman technique at Xi’an Jiaotong University. The crystals were cut along the desired orientations with the aid of X-ray diffraction (XRD), i.e. [100], [$01\bar{1}$]and [011] directions were assigned as *x*, *y*, and *z* axes, respectively. Poling of the crystal was performed using a homemade setup consisting of a sample holder immersed in a silicone oil bath and a high-voltage source (XTECH HV-20KV). Before poling, the samples were first cut to desired size and annealed at 700℃ for 5 h with short-circuit condition. The samples were then slowly cooled to room temperature to reduce the internal stress. PIN-PMN-PT single crystals with a domain size of 15 μm were obtained by using orthogonal poling method. The orthogonal poling method mainly contains two steps. Firstly, poling the tetragonal PIN-PMN-PT sample with an electric field (*E_1_*) of double coercive field along [011] direction at room temperature, the dwelling time is 5 mins. Secondly, applying an electric field (*E_2_*) on the “2T” sample along the [$0\bar{1}1$] direction. In our experiment, the intensity of *E_2_* is 1/5 of coercive field.

The [011]-poled PIN-PMN-38PT with domain size of 1-2 µm was used for SHG measurements and the sample size is 1.2 mm×1.2 mm×5mm ([100] ×[$01\bar{1}$] × [011]).

- 1. **Domain characterizations**

Optical observation of the domain structure was performed using a polarized light microscope (PLM) (OLYMPUS BX51, Japan) with a 0/90° crossed polarizer/analyzer pair. A Second harmonic generation (SHG) microscopy and polarimetry were performed using a modified Witec Alpha 300S confocal Raman microscope equipped with a 10 nm-resolution XYZ piezo-translation stage.

- 1. **Characterization of Nonlinear Optical Performance**

An optical parametric oscillator (OPO, Litron) was used as the pump source. The maximum pulse energy of the incident laser was about 63 mJ with a repetition rate of 10 Hz. The incident laser was focused by a convex lens with a focal length of 25mm and the radius of the focused spot was about 1 mm which was smaller than the sample but larger than the domains, and the diffraction effect cannot be neglected. Although the light generated by SHG is spontaneously separated from the FL, we still use a low-pass filter in front of the detector to prevent interference from the fundamental beam.

1. **Nonlinear optical intensity in single domain area**

The coordinate system is established with the spontaneous polarization direction of a single domain as the *z* axis, for example, x, y, *z* axis are [100], $[00\bar{1}]$ and [010] in a single domain (x, y, *z* axis are [100], $[001]$ and [01] in a adjacent domain). By following the Voigt notation, the nonlinear susceptibility matrix can be describe as in the 4mm sample:

$d_{ij}=\left( \begin{matrix} 0 & 0 & 0 \\ 0 & 0 & 0 \\ d_{31} & d_{31} & d_{33} \end{matrix}\begin{matrix} 0 & d_{15} & 0 \\ d_{15} & 0 & 0 \\ 0 & 0 & 0 \end{matrix} \right)$ (S1)

$\boldsymbol{P}_{i}^{2\omega}$depends on the optical susceptibility tensor ($d_{ij}$ elements) and on the electric field of the fundamental wave ***E*** (*E_x_* = 0, *E_y_*≠0, *E_z_*≠0)

$E_{x}\left( \omega\right)=0$ (S2)

$E_{y}\left( \omega\right)=E_{0}\cos\varphi$ (S3)

$E_{z}(\omega)=E_{0}\sin\varphi$ (S4)

Where φ is the polarization angle of the fundamental wave

$\left( \begin{matrix} P_{x}^{2\omega}(\varphi) \\ P_{y}^{2\omega}(\varphi) \\ P_{z}^{2\omega}(\varphi) \end{matrix} \right)=\varepsilon_{0}\left( \begin{matrix} \begin{matrix} 0 & 0 & 0 \\ 0 & 0 & 0 \\ d_{31} & d_{31} & d_{33} \end{matrix} & \begin{matrix} 0 & d_{15} & 0 \\ d_{15} & 0 & 0 \\ 0 & 0 & 0 \end{matrix} \end{matrix} \right)\left( \begin{matrix} \begin{matrix} 0 \\ E_{0}^{2}\cos^{2} \varphi\\ E_{0}^{2}\sin^{2} \varphi\end{matrix} \\ \begin{matrix} E_{0}^{2}\sin2\varphi\\ 0 \\ 0 \end{matrix} \end{matrix} \right)$ (S5)

Therefore, without analyzer, the SHG intensity is

$I^{SHG}\left( \varphi\right)\approx\left| P_{x}^{2\omega}\left( \varphi\right) \right|^{2}+\left| P_{y}^{2\omega}\left( \varphi\right) \right|^{2}+\left| P_{z}^{2\omega}(\varphi) \right|^{2}$ (S6)

In the adjacent domain, the polarization angle of the fundamental wave φ’ is φ-90°.

1. **Additional Figures and Tables**
   1. **Polarization Vectors in Tetragonal PIN-PMN-PT**


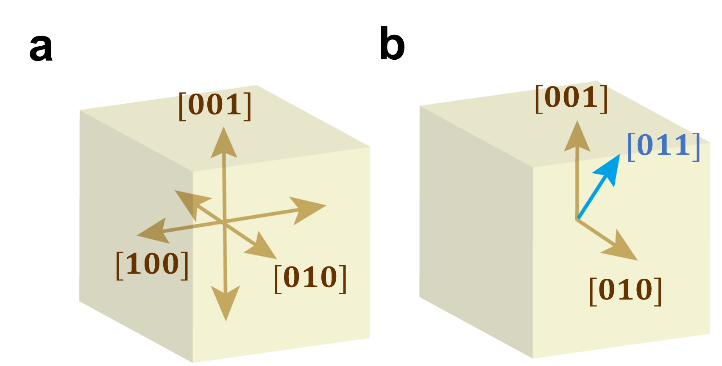


**Figure S1. Domain patterns and polarization orientation in [011]-poled tetragonal PIN-PMN-PT crystal. a,** Possible polarization vectors in as-grown (left) and **b,** [011]-poled (right) PIN-PMN-PT.

In the naturally grown tetragonal PIN-PMN-PT, spontaneous polarization vectors align with one of six equivalent <001> directions. After poling along the [011] direction, only two domain variants with polarization vectors along [010] and [001] existed forming a relatively simple "2T" engineered domain structure.

**3.2 Linear Optical Property**

**
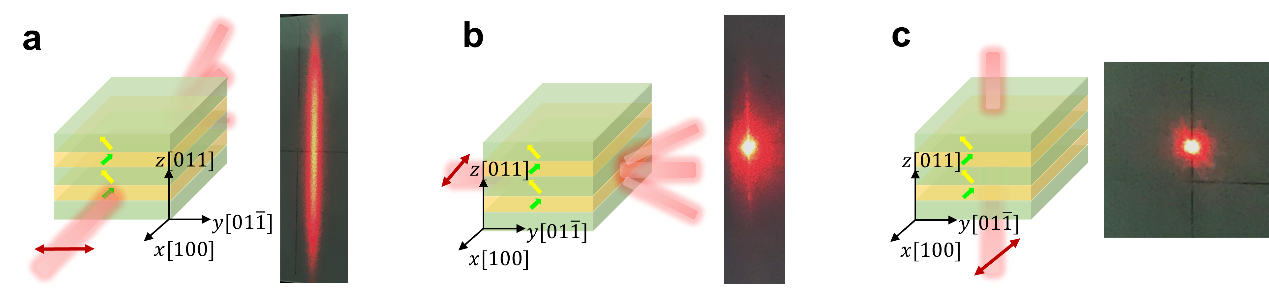
**

**Figure S2. Linear optical properties of domain-engineered PIN-PMN-PT crystals a, b,** and **c** Output spot of a Gaussian beam propagates along [100], [$01\bar{1}$] and [011] direction of the crystal, and the laser polarization is along the *y* direction.

The average domain size in the sample is a 2μm. While changing the polarization stare of the incident laser, the output spot of [100] and [011]-cut sample didn’t change. However, the deflection of the [$01\bar{1}$] cut sample is weakened.

**3.3 Nonlinear Optical Property**

**
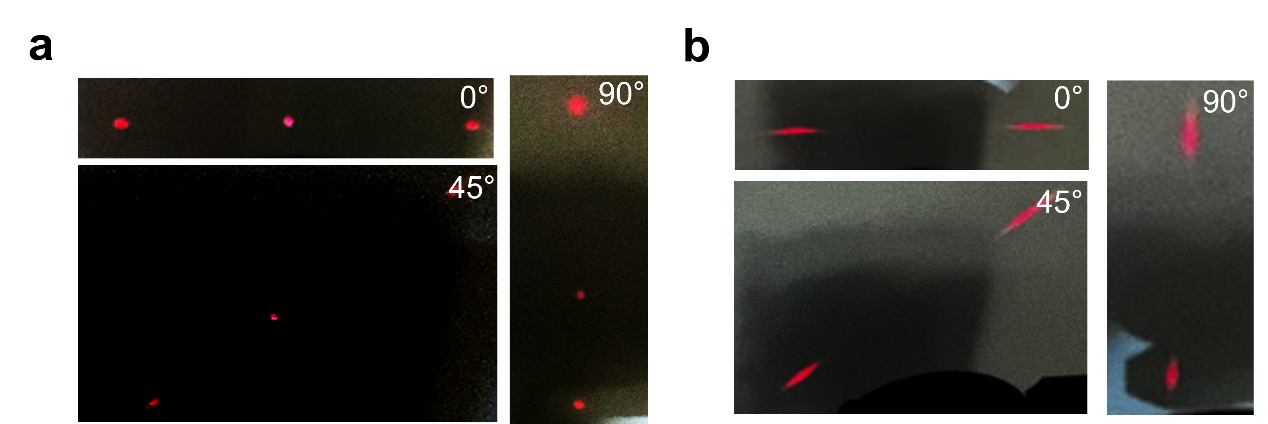
**

**Figure S3. SH intensity distribution image for different rotation angle of different cut samples. a and b** Output spot of a FL at 1200 nm propagates along [$01\bar{1}$] and [100] directions. The angle labeled in the upper right corner of each image is the angle between the polarization direction of the fundamental frequency light and direction of the sample.

In the initial state, the polarization direction of the fundamental frequency light is parallel to the [$01\bar{1}$] axis of the crystal, and its initial angle is defined as 0°. Then rotate the sample counterclockwise to change the angle between the [$01\bar{1}$]axis of sampleand the polarization direction of FL. The figures present the images of the SH spot in the initial state and with angles of 45° and 90°. It can be seen that when the relative position is changed, the distribution of the SH spot still follows [$01\bar{1}$].

**3.4 SHG processes in [**$\mathbf{011}$**]-cut sample**


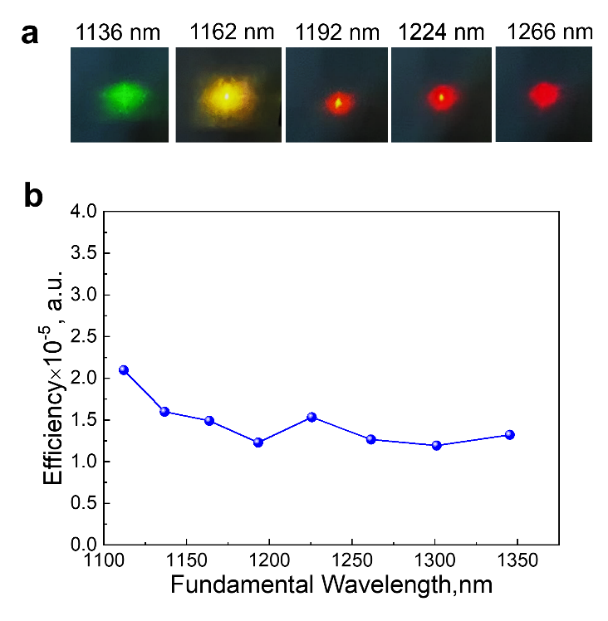


**Figure S4. SHG performance in [011]-cut sample. a.** SHG patterns from domain engineered PMN-PT samples at the FL wavelength from 1136 nm to 1266 nm. **b.** Dependence of the conversion efficiency on fundamental wavelength.
